# Creative reflections on embodied filmmaking: in, through and between the senses and spaces of the medicalized body

**DOI:** 10.1080/17458927.2024.2390729

**Published:** 2024-09-17

**Authors:** Olivia Turner

**Affiliations:** School of Arts and Cultures, Newcastle University, Newcastle upon Tyne, UK

**Keywords:** Creative practice, feminism, moving image, embodiment, medicalization, medical humanities, visceral body

## Abstract

This artist’s reflection further explores the imagined and sensorial encounters of the body in medicine through a creative practice-led feminist, embodied, and intersubjective approach to filmmaking. It uses “corporeal parenthesis” as form and content to destabilize conventional readings associated with medicalization. The sensations we feel on the inside of the body shape our imagined anatomical understanding. This extension of proprioception is a sensorial way of seeing and experiencing the inside of one’s own body. The visceral body, which entwines our real and imagined bodily inside, is positioned as a site for sense-making. This emphasizes knowledge as imaginative, intuitive, and lived to resist the standardization experienced within medicine. However, the passive horizontal body in the clinical encounter and the presumed intimacy of the therapeutic space, assumes the body to be accessible and consenting. This alters the ways in which we experience senses associated with pleasure and non-pleasure. By focusing on the artist’s moving image work *O (Symptom)*, creative practice is used to examine in, through, and between the spatial and sensorial boundaries of the medicalized body.

## Introduction

This reflexive piece of creative-critical writing centers the author’s own artwork and experiences. *O (Symptom)*^1^ encapsulates a body of artworks I created through performance, moving image, and sculptural installation. It explores the body in medicine through imagined sensorial and spatial clinical encounters. Here, the moving image aspect of this broader body of work is the focal point.^2^ Therefore, the title, *O (Symptom)*, used going forward, is referring only to the moving image artwork, which was screened at the Watershed in Bristol as part of the conference: *Senses in Modern Health/Care Environments: International and Interdisciplinary Perspectives*. Following the conference, this artwork and writing were invited as a creative reflection for this Special Issue, *Senses and Spaces of Modern Health/care*.

**Figure 1.**
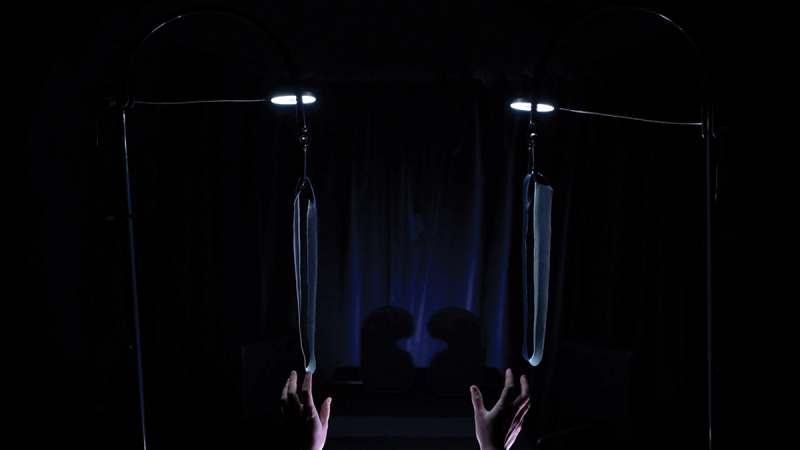
Still image from Olivia Turner, *O (Symptom)*, 2021, moving image artwork.

In *O (Symptom)*, there are three central acts that are staged within imagined pseudo-clinical rooms that, for me, feel unavoidably sequential from my experiences as an examined body: the waiting room, examination room, and dissection room. I designed and created these sets as interlinking spaces (Figure 1), suggestive of these experiences of being a body in medicine that invariably lead on from one to another Saunders (2008, 1–2). The associations between these spaces and sets are entangled with the clinical encounter, whereby the medical gaze shapes the power dynamic and the patient is silenced, reduced, and objectified Foucault (1973/1994, xiii-xvi). This creative work is situated within visual medical humanities and uses Fiona Johnstone’s notion of “productive doubt” Johnstone (2018) to unfix the assumed behaviors and gazes in medicine by favoring artistic embodiment and ambiguity.

The artwork is framed within a concept I call “corporeal parenthesis.” It uses the function of parentheses in an experimental way to reconceptualize how in between states coexist. They always have an opening and closing, an inside and outside. This requires a particular type of attention by the reader to examine the parenthetical space, analogous to the clinical encounter. The visual form of corporeal parenthesis demarcates physical and metaphorical openings and spaces into and around the visceral body. In view of this, it is important that the writing is understood as creatively coexisting with *O (Symptom)* through corporeal parenthesis.

The central narrator in *O (Symptom)* is a woman’s voice, in actual fact it is *my* voice, who speaks and sings about what is happening to the protagonist’s medicalized body. In the opening scene, her first words uttered are: “To conjure in you an O,”^3^ this marks the beginning of the examination. You, the viewer, assume the leading role in this artwork, for it is you and your body that matter most here. The narrator continues on and repeatedly sings the sounds “oh, oh, oh, oh, ooooaaaaoooooh,”^4^ as we move slowly through the staged set on the screen. The form of *O (Symptom)* is fragmentary, disorientating, and multi-voiced, mirroring the experience of illness and being a patient; in particular, the atemporality and aspatiality of chronic illness.

**Figure 2.**
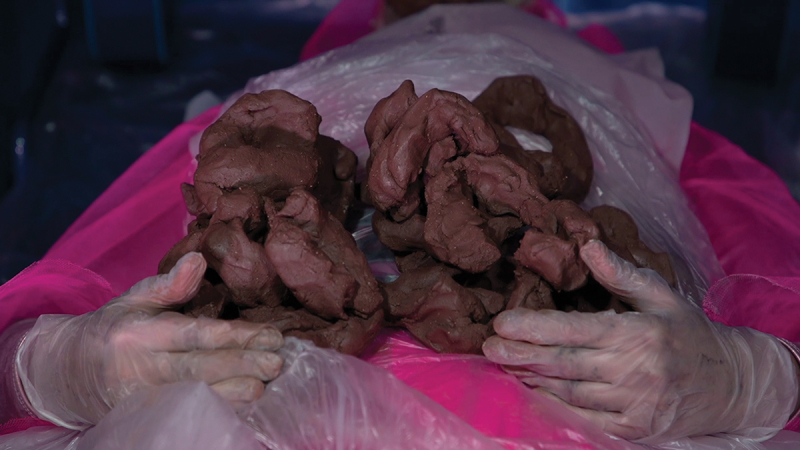
Still image from Olivia Turner, *O (Symptom)*, 2021, moving image artwork.

The premise of *O (Symptom)* is that *you*, the viewer and the “patient,” undergo an examination and procedure to see inside your body. The “doctor” climbs into your mouth, down your throat and into your stomach. As a result of the procedure, you become “Cadaver, the new sick,”^5^ and your body turns to clay (Figure 2). Sickness is the result of the clinical encounter, instead of preceding a requested examination. This visceral meeting point between the narrator’s body and viewer’s body is explored in the performance *Voicings* (2016) by artist, Florence Peake. Peake takes two pieces of clay: she places one on top of a table and pushes her fingers in to create two hollows that resemble eyes and calls “me,” and the other, she does the same but this time names “you.”^6^ Peake takes another piece of clay, holding it against her body and walks around the space, looking at each audience member whilst vigorously shaping the clay on her body. She states, “us.”^7^ When discussing her performance work with clay, she says, “How do you know you have a body? If you take away the visual, it’s through feeling and touch, pleasure and pain and sensorial experiences” Peake (2019). The merged clay forms and entanglement of “you” and “me” become an externalized part of Peake’s visceral anatomy, experiencing the physicality of the body, and placing the attention internal. The body turns into a site of material and imagination. Like in *O (Symptom)*, the sensorial meeting point between the protagonist and viewer’s body becomes slippery and porous – the intertwisting of *you* and *me* becomes a device to counteract the feeling of passivity as a medicalized body.

The visceral body, which encompasses our real and imagined bodily inside, is positioned as a site for sense-making. Whilst anatomical understanding of the medical body is instructed by scientific knowledge, the imagined interior is shaped by sensations, feelings and lived experiences Turner and Waite (2023). This brings to the fore the question of how an individual constructs a visceral image of their self and how it may change during circumstances of illness or disease Juler (2016). The disjuncture felt between the objectively “real” and subjectively imagined perception of our bodily insides is heightened when one becomes an examined body. As artist Carolyn Lazard notes, “there is a general consensus that the mind, in fact, resides within and is a part of the body. The material and immaterial parts of a person are enmeshed. Thoughts and feelings are tied to organs and tissues … There is a tension between reason and feeling that permeates illness and dominates most of what is living” Lazard (2019). Through a somatic film practice, *O (Symptom)* leans into this tension and the sensorial body is employed as a site of inquiry, expression and knowledge. Anne Cranny-Francis asserts that this way of making and viewing, “coerces the viewer to occupy a subjective position” Cranny-Francis (2009). By centering subjectivity, *O (Symptom)* emphasizes bodily knowledge as experiential, embodied, and lived to resist standardization and objectification within medicine. Therefore, the role of embodiment and haptic-sense-making are crucial for exercising one’s visceral autonomy in medicine.

The passivity of the supine body in the clinical encounter and the presumed intimacy of the therapeutic space, assumes the body to be accessible, compliant, and consenting Turner et al. (2023). The vulnerability of this position is also emphasized by the standing clinical staff present, who are literally and metaphorically standing over and looking down at you. Often it feels as though the difference between the land of the ill and the extremely ill is in some ways the degrees to which they are horizontal Sontag (1978, 3–4). The ill bodies are able to sit upright, propped by pillows, and retain the stature of their active body. The extremely ill are completely horizontal, like a parallel line drawn along the side of a hospital bed. As stated by the narrator in *O (Symptom)*, this horizontality of the patient is a position for “slipping between states.”^8^ It alters the ways we experience the senses normally associated with the supine body, slipping between feelings of touch, pain, pleasure, and non-pleasure; slipping between feelings of being patient and cadaver.^9^ This work explores through a feminist moving image practice, the ways the sensorial boundaries of the body are transgressed in medicine and the efforts to resist this.

The reduction of patient voice and authority were entrenched by the prevalence of pathologizing the living body through knowledge in the dissection room. Michel Foucault expands on this, stating “anatomy could become pathological only insofar as the pathological spontaneously anatomizes. Disease is an autopsy in the darkness of the body, dissection alive.”^10^ This objectification of the body saw the development of a visual and linguistic discourse Turner (2023, 64–65). The healthy body, ill body, and dead body became interchangeable and slippery states. As opposed to ways of being that are fundamentally opposed to one another and requiring separate types of learning and understanding. Within *O (Symptom)*, this interchangeability of the visceral body in medicine is developed through imagined interactions with and slippages between the doctor, patient, and cadaver.

The published US feminist health manifesto, *Women and Their Bodies* in 1970 (later revised as *Our Bodies, Ourselves*), addressed the ways medicine’s treatment of women resulted in the “alienation of us from our own body” Boston Women’s Health Collective (1970, 7). This pamphlet empowered women with knowledge that had been previously mediated by doctors. Stating its intention to “become more autonomous human beings, how we could act together on our collective knowledge to change the health care system for women and for all people” Boston Women’s Health Collective (1970, 4). However, the call to action still persists: Annabel Sowemimo’s recently published *Divided: Racism, Medicine and Why We Need to Decolonise Healthcare* highlights the racist patriarchal practices that continue to be entrenched within Western medicine. She stresses how she had to write this book because “health disparities, which have long persisted, reflect the social divisions, which seem set to worsen over the next few years” Sowemimo (2023, 327). In Helen Molesworth’s essay, *How to Install Art as a Feminist*, she asserts that feminism “is the fundamental reorganisation of the institutions that govern us” Molesworth (2010). Therefore, through an embodied feminist voice and creative practice, we can examine the visceral body and the way it is governed in medicine, to rethink and reimagine clinical encounters and spaces.

“Corporeal parenthesis” uses the function of parentheses in an experimental way to consider the metaphorical and physical relationships between writing, voice, touch, sight, feelings, and corporeality. Typically, parentheses create space within a sentence for in between states to coexist and are used to enclose additional information or intimate asides. They always have an opening and closing; an inside and outside. This requires a particular type of attention by the reader to examine the parenthetical space, analogous to the type of attention used to examine the body in medicine.

Through the development of imaging technologies and their relationship to the medical gaze, artist Liz Orton observes in *Becoming Image*, “To be a patient is increasingly to become an image” Orton (2018, 6). Historically, the transformative power of the gaze has been through anatomical drawings and atlases, whereby the image of the body and its textual quality become something to be *read*. Whilst these books serve as constructions and representations of knowledge, it is argued they draw the materiality of the body into a language in and of itself Albano (2001). This can be seen in anatomical atlases, like Johann Remmelin’s *Catoptron Microcosmicum*: the hinged skin of the figure on the page is lifted like a piece of cloth or fascia peeled back to reveal the bodily interior Remmelin (1691). The textual associations of the body-image in medical atlases depict a passive surface to be read.

Parentheses can also rupture and displace the movement and meaning of language. This enables ways to rethink and reimagine corporeal encounters and spaces. The sense of rupture and displacement is also inherent in chronic illnesses which, by their definition, exclude recovery and evade curative capabilities. When considering the medicalized body and its sick role, chronic illnesses are expected and required to be contained Parsons (1964, 261–262). However, by its very nature, chronic illness is a form of corporeal slippage – uncontainable, atemporal, and aspatial Kafer (2013, 25–35).

But as much as it is about *you*, this is also about *me*. As an artist living with chronic illness, *O (Symptom)* blurs my body with yours through an intersubjective approach. A central concern within autobiographical work is the relationship between “personal narratives” and “public stories,” and between narrator and audience, which are situated between fact and fiction Cosslett, Lury, and Summerfield (2000, 2). As acknowledged by Tess Cosslett, Celia Lury, and Penny Summerfield, the autobiographical stance connects personal and political, women’s experiences, and gendered subjectivities to become a “vital resource in the creation of women’s knowledge” Cosslett, Lury, and Summerfield (2000, 3). Autobiographical practice is inherently intersubjective, inclusive of written, spoken, and visual methods of storytelling, remembering and retelling, as explored within this work Cosslett, Lury, and Summerfield (2000, 2–5).

There are few areas in our lives not influenced by medicine and, for this reason, it feels important to locate the “I” in this work. The artist Carolyn Lazard writes on this process, “Everything that I have ever lived is concentrated in my cells and somehow persists even as my body continues to regenerate itself. This undifferentiated mass of tissue and memory, alive and sticky, is an unknown place worth approaching with an openness and willingness to let it reveal itself to me.”^11^ I have been personally confronted by medicine through various experienced moments and clinical interactions during the creation of *O (Symptom)* and because of the nature of this intersubjective approach, these moments and others in my life become inextricably linked due to their physical and emotional impact on me. These are *my* experiences, some more explicit and others implicit, which have come to shape a great deal of this artwork and writing.

## Corporeal parenthesis (sensorial slippage)

The visual form of “corporeal parenthesis” manifests as a sequence of near-touching curves that revolve around the shape of a circle. This delineates physical and metaphorical openings and spaces into and around the visceral body. Through this creative method, a sensorial and embodied approach to filmmaking is formed. Beyond their physical and functional status, an interesting aspect of parentheses is what Roi Tartakovsky refers to as their “semantic in-between-ness” Tartakovsky (2009, 215). Parentheses are borders between: text and grammar, sign and voice, looking and feeling, inside and outside, intimate and impersonal.

Through this semantic and corporeal in-between-ness, parentheses can be thought of as *extratextual*, able to exist outside of, adjacent to, on top of, or independent of text. They are, in this way, “fuzzy, punctuation marks [that] are amenable to appropriation, to exploitation, and to projection” Tartakovsky (2009, 216). E. E. Cummings plays with this semantic in between, as he “manipulates spatial, visual and syntactical elements of language as *material*” Webster (1995, 120). It is in this vein that corporeal parenthesis is used as a *material* within my creative practice.

Gertrude Stein was also experimental in her interpretation and exploitation of “fuzzy punctuation,” describing and personifying characteristics and behaviors: “commas are very servile, they have no life of their own they are dependent upon use and convenience” Stein (1971). Stein imbues punctuation with a personality and corporeality that brings it into relationship with the body. Even though parentheses are only visible within the written medium, its connection between punctuation and spoken or performed language has long been traced Baron (2001). This signifies the movement of parentheses off the page and into the mouth, to be regurgitated out through the body and into the voice. It is indicative of the slipperiness between form and content, speaking and singing, and analogous to the examined visceral body.

The voice and mouth are explicit sensorial sites. As Brandon LaBelle acknowledges in *Lexicon of the Mouth*, “the voice is precisely that which remains in a dynamic state, tensed between presence and absence, phonic, and textual substance, and driven by the pressures and pleasures of being a body” LaBelle (2014, 12). In *O (Symptom)*, the pressures and pleasures of voice and body are used as methods of resistance and critique within the context of medicine. The moving image work is fully scripted, describing the woman’s body as she moves through a sequence of imagined clinical spaces. Her body gradually twists into a site of demonstration. She enacts various types of medical examinations and investigations of the body, *your* body.

The voice moves between coherent poetic monologues to guttural frenetic vocal expressions. The repeated “Oh’s” mouthed by the singing voice are on the limits of language and the articulate. Language functions in clinical communication as a concise provider of information with an apathetic tone. The “Oh” in *O (Symptom)* slips between an expression of pain and pleasure, between empowerment and control of the body. The penetrating nature of voice is played with, and the expressive vocal soundings and narrated scripts threaten to transgress the boundaries of *you*. A visceral voice of and from the body is used to express corporeality beyond words.

Corporeal parenthesis is used as a framing device that moves through different metaphorical and physical iterations of a formal circular shape: whereby (), becomes an “O,” becomes an orifice. Corporeal parenthesis demarcates real and imagined openings and sensorial sites of the body. A continual shifting between corporeal states and forms. Georges Bataille also explores folding, refolding, and unfolding of form and content within writing and its relationship to objects. He accentuates the verges of the corporeal body and textual body.

This emphasis on the formal and sensory slippages of the circle was born from *Story of the Eye*:

two ancient and closely associated obsessions, *eggs* and *eyes* … I had absolutely no idea of what the glands I was writing about were really like, and [my friend] promptly read aloud a detailed description in an anatomical textbook. I thus learned that human or animal balls are egg-shaped and look the same as an eyeball. (Bataille 1928, p. 71)

Bataille’s *Story of the Eye* is also about a circular object, whose shape morphs and overlaps from one associated object to the next. This movement through different iterations of the circle renders form formless ⎯ the eye becomes a testicle, which becomes the sun. For the reader, his writing feels like you are falling between the gaps of form and meaning, unable to grasp what is right in front of you, even though the formal focus of the circle stays the same. Jean-Luc Nancy explains this quality as corporeal, “Bodies always about to leave, on the verge of a movement, a fall, a gap, a dislocation” Nancy (2008, 33). This, too, relates to our sensorial experiences, contingent, and fleeting, they are always on the verge of movement Mário Grilo (2014).

Drawing on Bataille and Nancy in this artwork, the corporeal matrix of parenthetical forms revolves around the circular shapes of the medical body. This shape not only falls between eyes, orifices, and openings but is a shape symptomatic of the pathological body, like tumors and cysts. The etymology of the word *symptom* is indicative of this slipperiness of form and content. Whilst the most recognizable appearance of the word is from the late Latin *symptoma* or “a happening, accident, disease,” this most interestingly stems from the Greek word *sympiptein*, meaning to “coincide, fall together” Online Etymology Dictionary (2020). Again, we are confronted by a falling sensation, aroused by the ungraspable textual and corporeal qualities exploited here in language and form. Explored in this moving image work, *O (Symptom)*, the symptom of *(O)* is a linguistic and vocal slippage that falls between lumps, tumors, sensations, dysfunctions, and deformations of the medical body.

*My clammy hands and flushed fingers*
*reach*
*between hot legs to feel*
*a cold metallic touch*.
*She’s whet*,
*the nurse, its blade⎯*
*curved like the edge of a duck’s bill*,
*to open and close an O*.
*(Oh) she could only apologise*
*for the pain*.

## Medical materiality

The sensory materiality of the medical body and medical spaces is explored through the costume and sets in *O (Symptom)*. When an individual attends a clinical appointment, they cross an institutional threshold that alters their personhood by exerting power and ownership over them, the senses are both heightened and numbed. There are distinct spaces encountered within the clinical setting, such as reception areas, corridors, waiting rooms, consulting rooms, examination rooms, changing rooms, and wards. As poet, performer, and writer, Jamie Hale outlines, “our agency is stripped along with your clothes and you’re given a matching gown, bedding, bed. Everyone looks the same” Hale (2020). This uniformity of the environment and medical materiality ruptures a person’s sense of agency. For certain procedures, patients are given a hospital gown to wear. Worn back-to-front and tied at the neck, the gown is left open at the back for ease of access to the body. By wearing the gown, assuming uniformity, and becoming “patient,” you and your body are presumed to be open and unobstructed for clinical staff.

**Figure 3.**
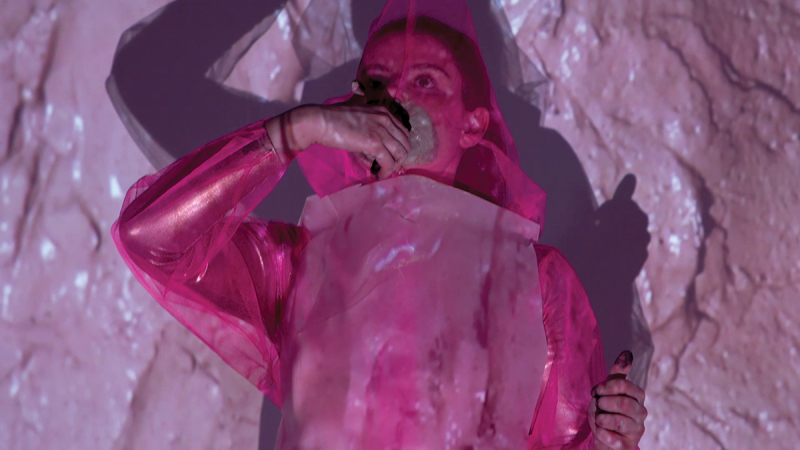
Still image from Olivia Turner, *O (Symptom)*, 2021, moving image artwork.

In *O (Symptom)*, the main figure wears a translucent plastic gown that remains open at the back, evocative of the dignity gown (Figure 3). This parenthetical shape created by the opening of the gown becomes a new “O,” an oval entrance to the body. Underneath, the protagonist wears two overlaid pink bodysuits, the contrast between the matt and shiny fabrics are suggestive of dry and moist, living and dead states of skin. The costume becomes a metaphor for the medicalized body and what Steven Connor terms, “the abject frailty and vulnerability of the skin, and the destructive rage against it exercised in violent fantasies and representations of all kinds” Connor (2004, 9).

Eve Kosofsky Sedgwick likens medical rooms to “punishment spaces,” whereby “it’s like the way people wait, about to be punished, in my fantasies. Names on the intercom, summonses to a fate, slash at the fragile pretense to privacy” Kosofsky Sedgwick (1999, 48). It is not only through meaning and experience conjured by the spaces, but the language used to describe them, which for Kosofsky Sedgwick, is just as visceral: “Doors open and doors close; people peer in. The ‘examination:’ fearful word!” For Kosofsky Sedgwick, language and spaces within medicine acquire a liminality, binding together disparate experiences to make sense of the chaos and alienation she feels as a medicalized body.

However, Kosofsky Sedgwick also embeds themes of clinical companionship and care within her writing. She uses the metaphor of a bath to describe the space she shared with her therapist, Shannon: “a kind of tepid bath” Kosofsky Sedgwick (1999, 66). She admits, “Besides, I like *patient*. It is true I can be very patient. And Shannon is like this too, so the word doesn’t feel like placing me at a distance” Kosofsky Sedgwick (1999, 13). The welcomed adoption of the role of patient and holding space of the therapist’s room is transformed into a positive sensory environment. She has “splashed around so happily in his mild, hired companionship” Kosofsky Sedgwick (1999, 51). The tepid characteristic of this space is reflected in Kosofsky Sedgwick’s pleasure of “being *impersonally* held” Kosofsky Sedgwick (1999, 67). The anonymity of architecture, the relationship between patient and doctor, and the language used become transformative and generates intimacy, like the parenthetical space. The clinical encounter is not a fracturing or disorientating experience for Kosofsky Sedgwick. Instead, the metaphorical bath, “into which I/slowly lower my great bulk,/to be supported” Kosofsky Sedgwick (1999, 68), has a safe elasticity that is accommodating of the patient, in all their multiplicity of subjective experiences.

**Figure 4.**
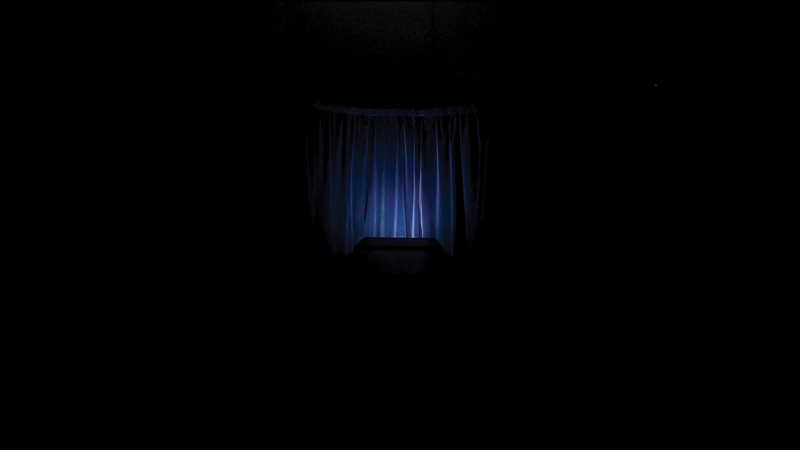
Still image from Olivia Turner, *O (Symptom)*, 2021, moving image artwork.

Intimate sensorial spaces are also explored using the curtain in *O (Symptom)* (Figure 4). In these various contexts, the curtain is a liminal object: a portal or marker of the in between. It delineates a person’s private space from public space, between audience and performer, reality, and imagination. Within the medical context, the curtain is made of a lightweight and unsubstantial cloth material. This thin, membranous material mirrors the fragile attempt of privacy for the patient. Like the patient’s body, their therapeutic space is assumed to be always open and unobstructed.

The curtain featured in *O (Symptom)* is made from a heavy blue velvet, contrasting the sterile and wipeable materials found within the medical context. This curtain is domestic, from my own family home, shifting the evocation of the object into an even more personal and intimate space. It rubs up against material and conceptual suggestions of the medical examination table, set within the same scene. The curtain is also threefold: the privacy curtain drawn around a hospital bed; the theatrical front curtain opened and closed during a performance to reveal or conceal and commence or conclude the stage; and the curtain closed on life in death. The supporting metal frame is shaped in a semi-circle with a pair of curtains side by side, like a pair of parentheses, enclosing the space around the bed. The parenthetical motif is used to link objects, form, and grammar. It is as if the rest of the sets and film is a sentence: the curtain-parentheses demarcate a separate but interconnecting space; an intimate aside.

## Conclusion

Through an intersubjective and sensory approach to moving image work, *O (Symptom)* explored and reflected on the visceral body in medicine. It has drawn together contemporary feminist artistic practice into dialogue with medical humanities, medicine, and medicalized corporeality. By also undertaking research that is speculative and experimental, it favors “an openness to accident and serendipity, spontaneity; artistic risk, emotional urgency and intensity” Shields (2010, 3), which is fundamental to practice-led research. Moving image, like lived experience, is untethered and cannot be held still. So instead, it leans into slipperiness as a method for challenging the horizontal, passive body that so often I have become within medicine. Moving image enables an alternative form of visual language to speak to these experiences, one attuned to embodied material and sculptural concepts and methods, beyond conventional approaches.

This artist reflection draws on a multiplicity of slippery definitions and manifestations of the visceral body, navigated through real and imagined clinical encounters between the doctor, patient, and cadaver. It has studied and critiqued the different types of senses, languages, and conditions “acted upon” Parker-Starbuck (2016) the body in medicine. As James Elkins argues, “There is no such thing as just looking.” Sight within medicine is entwined with the objectifying and intrusive vision. Instead, visuality in moving image establishes a dynamic of spectatorship and, by implicating or “coercing” the viewer’s body, ethics of looking and seeing Mermikides and Bouchard (2016, 4). *O (Symptom)* brings attention to the distinct forms of intervention and critique that visual arts can contribute surrounding health and medicine.

*O (Symptom)* has been screened nationally and internationally. Often these screenings take place in dimly lit cinematic spaces. I recently went to hospital to attend an appointment for a procedure that involved medical imaging. I was ushered into a small room that was dark inside, lit only by monitors that acted as my guiding light. It feels important to recreate the experience of that space in some capacity for the viewer. In that moment, my insides were presented back to me on a screen and my body began to read like it was unfixed and indeterminate. The nature of dark spaces always evokes a sense of intimacy, whether you are by yourself or with another. I think it is because you are no longer reliant on your sight as your dominant sense to guide you and this can make you feel vulnerable. Being within a hospital makes you feel even more vulnerable, which in turn can make you a passive and compliant patient.

In my experience, often the patient is transmuted into an image through medical visualization Casini (2021, 190–192). For example, undergoing an x-ray examination, whilst the body is not physically transgressed, unlike surgery, does the absence of physical touch mean that the bodily boundaries remain untouched?^12^ Catherine Waldby argues, “the condition of [the medical practitioner’s] visual access to the body’s interior is precisely the introduction of some change into the body … In other words the body has to be ‘touched’ or transgressed in some way so that it can be envisaged” Catherine Waldby (1997). In reference to the penetrative medical gaze of noninvasive technology, Liz Orton states, how “medical images, with their promise to visibilise the unseen, seem to violate emotional and psychological boundaries.”^13^ The real-time image capturing of your bodily interior is unique and deeply personal. Whilst this might be a more accurate and true representation of your insides than the anatomical model, a disjuncture forms in how you feel and imagine your insides. The dissociative state and passivity experienced in the clinical encounter acts as a coping mechanism for transgressive experiences, whereby your body no longer looks or feels like your own.

Seeing through skin to visualize the bodily interior is, in itself, a way of “touching” the body, bringing into direct correlation the hand and eye. James Elkins admits this objective modality of looking “is also inconstant seeing, partial seeing, poor seeing, and not seeing … seeing is also blindness” Elkins (1996, 95). When discussing the physical limitations of our eyes, a visuality *of* and *from* the body provides space for an imaginary gaze to challenge these limitations. By creatively opening out medical senses beyond the penetrative clinical gaze, an exploration of the slippages between real and imagined materiality and corporeality can occur.

The emergence of creative practice-led research in the field of critical medical humanities has opened new ways undertaking research that are speculative and experimental. This entanglement with the “visual turn”^14^ in medical humanities, brings a distinct contribution by directly exploring the intersection of contemporary fine art, moving image, and health. The public staging of this work, as Ludmilla Jordanova states, brings the body and its relationship with medicine to be “opened up for critical inspection” Jordanova (2014, 61). This brings to attention the novel forms of intervention and critique the visual arts can contribute surrounding health and medicine.

## Notes

1. Olivia Turner (2021) *O (Symptom)* [Performance, installation and moving image].

2. A short excerpt of Olivia Turner’s moving image artwork *O (Symptom)* (2021) is available at: https://www.oliviaturner.co.uk/o-symptom-film.

3. *O (Symptom)* (2021) Directed by Olivia Turner [Moving image artwork].

4. Ibid.

5. Ibid.

6. Florence Peake (2016) *Voicings* [Performance. The Cockpit, Serpentine Galleries. 14 May].

7. Ibid.

8. *O (Symptom)* (2021).

9. Turner et al. (2023) “Reimagining Medical Encounters and Intimacies,” pp. 64–65.

10. Foucault (1973/1994) *he Birth of the Clinic*, p. 131.

11. Lazard (2019) “The World Is Unknown.”

12. Saunders (2008) *CT Suite*, pp. 2–10.

13. Orton (2018) *Becoming Image*, p. 5.

14. Johnstone (2018) “Manifesto for a Visual Medical Humanities.”
